# Soil quality and microbial communities in subtropical slope lands under different agricultural management practices

**DOI:** 10.3389/fmicb.2023.1242217

**Published:** 2024-01-08

**Authors:** Ching-Nuo Chen, Chien-Sen Liao, Yu-Min Tzou, Yu-Te Lin, Ed-Haun Chang, Shih-Hao Jien

**Affiliations:** ^1^Department of Civil Engineering, National Pingtung University of Science and Technology, Neipu, Taiwan; ^2^Department of Biological Science and Technology, I-Shou University, Kaohsiung, Taiwan; ^3^Department of Soil and Environmental Sciences, National Chung Hsing University, Taichung, Taiwan; ^4^Department of Soil and Water Conservation, National Pingtung University of Science and Technology, Neipu, Taiwan; ^5^MacKay Junior College of Medicine, Nursing and Management, Taipei, Taiwan

**Keywords:** land degradation, agricultural management practice, soil carbon stock, microbial community, phosphorous-containing herbicide

## Abstract

Land degradation is a major threat to ecosystem. Long-term conventional farming practices can lead to severe soil degradation and a decline in crop productivity, which are challenging for both local and global communities. This study was conducted to clarify the responses on soil physicochemical properties and microbial communities to changes in farming practices. Slope land orchards under three agricultural management practices—conventional farming (CF), organic farming (OF), and ecofriendly farming (EFF)—were included in this study. We found that soil carbon stock increased by 3.6 and 5.1 times in surface soils (0–30 cm) under EFF and OF treatments, respectively. EFF and OF significantly increased the contents of total nitrogen by 0.33–0.46 g/kg, ammonia-N by 3.0–7.3 g/kg, and microbial biomass carbon by 0.56–1.04 g/kg but reduced those of pH by 0.6 units at least, and available phosphorous by 104–114 mg/kg. The application of phosphorous-containing herbicides and chemical fertilizers might increase the contents of phosphorous and nitrate in CF soil. High abundances of *Acidobacteria* and *Actinobacteria* were observed in EFF and OF soils, likely because of phosphorous deficiency in these soils. The abundance of fungi in OF soil indicated that plants’ demand for available soil phosphorous induced the fungus-mediated mineralization of organic phosphorous. High abundances of *Gammaproteobacteria*, *Planctomycetes*, *Firmicutes*, and *Nitrospirae* were observed in CF soil, possibly because of the regular use of herbicides containing phosphorous and chemical fertilizers containing high total nitrogen contents.

## Introduction

Land degradation is a key global concern that requires immediate attention. Sustainable soil management practices may mitigate the effects of climate change (Lal, 2004) and effectively resolve the problems associated with increasing world population and food insecurity (Gnanavelrajah et al., 2008; Lal, 2018). For sustainable soil management, soil quality must be preserved, particularly by augmenting soil organic stock and biodiversity through various agroecological practices, such as the implementation of organic farming (OF) practices, the reduction of chemical input, and the enhancement of plant diversity through crop rotation or agrisilviculture (Van Wesemael et al., 2010). Furthermore, increasing the organic carbon input into cultivated soils has considered as a main option for reducing their negative climate effect, and even reversing it by turning cultivated soils into a net carbon sink through soil organic carbon (SOC) sequestration (Amelung et al., 2020).

Food demands and land shortage bring intensive farming and overuse of chemical fertilizers in farmland soils in Taiwan. Under tropical and subtropical climates, SOC contents are generally lower than 20 g kg^–1^ (Huang, 1994) in farmland soil in Taiwan. In order to mitigation of climate change and restoration of agroecosystem, OF or eco-friendly farming (EFF) have received great attention from researchers investigating the sustainability and productivity of agricultural soils. As we known, conventional farming (CF) often involves the overuse of chemical fertilizers and herbicides, which accelerates soil degradation and poses environmental risks. By contrast, OF or EFF involve the use of organic fertilizers, which enhance crop yield by increasing the content of organic matter in soil (humus) and soil quality. This increase in organic matter content enhances soil biological activity and improves soil structure, water infiltration and retention, and nutrient storage (Peltoniemi et al., 2021). To clarify the effects of management practices on soil quality and microbial communities should be the critical step to mitigate soil degradation and food security due to climate change.

Besides SOC stock, bacterial communities are key players in soil ecosystems because they mediate many soil functions and processes, such as organic matter decomposition, nutrient cycling, and nitrogen fixation (Bender et al., 2016). The efficiency of nitrogen utilization is dependent on the abundance of soil bacteria involved in nitrogen fixation and nitrification (Levy-Booth et al., 2014). The diversity of soil bacteria may serve as a bioindicator of soil quality (Pereira e Silva et al., 2013). Several studies have revealed that agricultural practices alter the composition, diversity, activity, and biomass of soil microbes. The composition of a soil bacterial community varies between the OF and CF systems; microbial activity as well as biomass carbon and nitrogen contents are higher under the OF system than under the CF system (Peltoniemi et al., 2021). The abundances of soil bacteria associated with carbon, nitrogen, phosphorous, and sulfur cycles have been reported to be enhanced under the OF system (Zhang et al., 2019). In addition to the observed changes in soil composition, OF resulted in elevated crop yield; furthermore, OF positively affected soil pH, calcium contents, phosphorous content, and enzymatic activity (Durrer et al., 2021). To understand the effects of agricultural management practices on the soil ecosystem, comprehensive studies must be conducted on the changes in soil physicochemical properties and microbial communities under various agricultural management practices.

Therefore, this study was conducted to (1) simulate and understand the spatiotemporal variations in the responses of SOC and microbial communities to changes in farming practices, (2) clarify the associations between agricultural management practices, soil properties, microbial diversity, and soil organic carbon stocks, and (3) evaluate the mechanisms which how do SOC stock change with soil properties and microbial communities affected by different management practices.

## Materials and methods

### Study area

**FIGURE 1:**
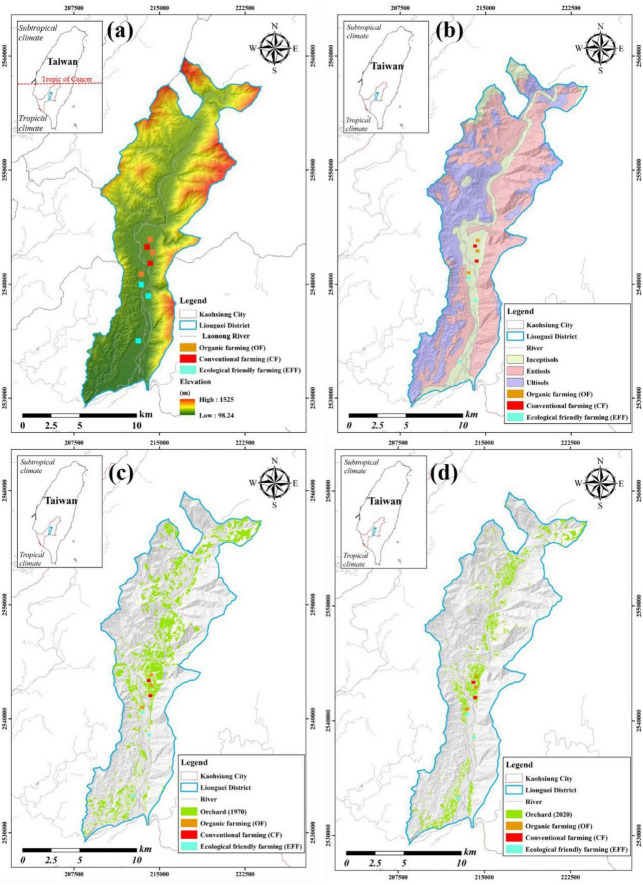
Study sites (orchards) under various agricultural management practices **(a)** elevation and distribution of studied orchards; **(b)** soil orders distribution in Liouguei district; **(c)** land uses (only orchards shown in green color) in 1970; and **(d)** land uses (only orchards shown in green color) in 2020.

This study was conducted in eight orchards where mango (*Mangifera indica*) is the dominant planting fruit at these orchards. These orchards are located at an alluvial riverbank in Liouguei district (23°00′N; 120°39′E) in Laonong River catchment, southern Taiwan. The climate in our study area is topical climate with mean annual rainfall of >2,000 mm and an annual temperature of 25°C. The soils at our studied orchards belong to the same soil type which is characterized by slightly acidic and low fertility. The soil could be classified as Typic Haplustept based on Soil Taxonomy (Soil Survey Staff, 2016). This soil (Inceptisol) is characterized by sandy loam texture, shallow soil depth and weak weathered degree (Figure 1).

Since 1970, conventional farming (CF) practice has persistently carried out at these eight studied orchards in this study (Figure 1). However, among these orchards, three were converted to organic framing (OF) from CF since 2010, and another three one were converted to eco-friendly farming (EFF) from CF since 2017. In the orchards under the CF system, the farmers applied two commercial chemical fertilizers. One fertilizer (application rate, 2 tons/ha/year) comprised 26% total nitrogen (TN), 13% phosphorus pentoxide, and 13% potassium oxide, whereas the other (application rate, 1.4 tons/ha/year) comprised 14% TN, 28% phosphorus pentoxide, and 14% potassium oxide. In addition, CF involved the biannual application of the herbicide (*RS*)-2-amino-4-(hydroxy-methyl-phosphoryl) butanoic acid (glufosinate). In the grass-covered orchards under the OF system, soybean meal compost was applied as an organic fertilizer (application rate, 50 tons/ha/year). In the grass-covered orchards under the EFF system, chicken manure and soybean meal composts were applied (application rate, 20 and 2 tons/ha/year, respectively). No herbicide was used under the OF and EFF systems.

### Soil sampling and analyses

Soils (about 2 kg) were collected in depth of 0–15 cm, 15–30 cm, and 30–50 cm, respectively at each orchard under different management practices in 2020. Soil sampling plot at each orchard were randomly selected in five replicates. Soil samples were collected from the different soil depths of each sampling plot, air-dried, gently ground to pass a 2-mm sieve, and used for the analysis of physical and chemical properties.

The pH values of soil samples collected from the study orchards were determined in a mixture with deionized water [1:2.5 (w/v) for soil]. The Horiba F-74 BW meter was used for measuring pH values (Rhoades, 1982). Soil particle-size distribution was assessed using the pipette method (Gee et al., 1986). Cation exchange capacity was evaluated using the ammonium acetate (pH 7.0) method (Sumner and Miller, 1996). Exchangeable potassium was extracted with 1 mol/L ammonium acetate solution [1:10 (w/v) for the soil samples; 1:20 (w/v) for the biochar samples] and assessed through atomic absorption spectrometry (Z-2300; Hitachi, Japan). The SOC content was measured using the wet oxidation method (Nelson and Sommers, 1996). The content of available phosphorous was measured using the Bray P-1 test (Bray and Kurtz, 1945). The contents of ammonia (NH_4_^+^-N) and nitrate (NO_3_^–^-N) were measured through steam distillation performed using magnesium oxide and Devarda’s alloy (Mulvaney, 1996).

The content of SOC stock was calculated as follows:


$$\text{SOC}=B\,d_{i}\times C_{i}\times \left(1-S\,t\,o\,n\,i\,n\,e\,s\,s_{i}\%\right)\times {\text{t}}_{i}$$

where SOC is the carbon stock in soil up to a depth of 30 cm (kg/m^2^), Bd_*i*_ is the bulk density of the soil (mg/m^3^) in layer *i*, C_*i*_ is the content of carbon in the soil (milligrams of carbon per gram of soil) in layer *i*, stoniness_*i*_% is the percentage of rock fragment in soil collected from a depth of > 2 mm, and t_*i*_ is the thickness of layer *i* (depth = 0–30 cm). The stoniness percentage was estimated in the field. In this study, five replicates (about 200 gram soil were collected for each replicate) were sampled in each studied orchard with different management practices. The SOC stock (kg/m^2^) in 0–15 cm, 15–30 cm and 30–50 cm, and SOC sequestration rate (kg/m^2^/year) in surface soil (0–30 cm) were also calculated in this study to evaluate the benefits of OF and EFF on SOC stock compared with CF in this study.

### Modeling of environmental variables for SOC stock prediction

This study used rainfall, air temperature, elevation, slope gradient, vegetation index, clay content, bulk density, rainfall erosivity factor (Rm) and erodibility factor (Km) as predictors of SOC in the selected study area (Liouguei district) in southern Taiwan. Meteorological dada including rainfall and air temperature during (2010–2022) were collected from Taiwan Central Weather Bureau. Elevation and slope gradient obtained from digital elevation model (DEM) were downloaded from Taiwan government open data platform.^1^ Normalized difference vegetation index (NDVI) was calculated from LANDSAT 8 data from USGS. Soil properties such as clay contents and bulk density were obtained from soil survey data base of Taiwan Agricultural Research Institute (TARI). The Rm and Km maps were downloaded from slopeland info express from Taiwan Soil and Water Conservation Bureau.

All the models were developed using Cubist regression, and executed using the R-4.3.1 for windows statistical software^2^ with the Cubist (Version: 0.0.18) package (Kuhn et al., 2023). Cubist is a powerful data-mining tool for generating rule-based predictive models from data. Generally, a Cubist regression model has a good predictive power and is also easy to understand and interpret (Minasny and McBratney, 2008). This data-mining tool can also apply boosting-like scheme called committees where iterative model trees are created in sequence. The details of the boosting procedure can be found in Quinlan (1992). Furthermore, the Cubist model also provides the attribute usage (relative importance) of each variable which indicates the importance of the variable in the model.

In R 4.0.5, a random sampling method was employed to divide the dataset into a 70% training set (calibration set) and a remaining 30% validation set. The model’s performance was evaluated using the root mean square error (RMSE) and the coefficient of determination (R^2^) calculated from the validation set. In this study, a dataset of soil properties were collected from detail soil survey project (Taiwan SSURGO database) carried out by TARI during 2010 to 2015, about 225 topsoil (0–30 cm) data were used in this study.

### Analysis of phospholipid-derived fatty acids

Phospholipid-derived fatty acids (PLFAs) were extracted and analyzed following a method described by Frostegård et al. (1993). PLFAs were extracted using a single-phase mixture of chloroform–methanol–citrate (1:2:0.8). Fatty acid methyl esters were analyzed through capillary gas chromatography and detected through flame ionization by using the Thermo Finnigan Trace chromatography system, as described by Koza and Rezanka (1989). Fatty acid nomenclature was performed as described by Frostegård et al. (1993). The cumulative content of PLFAs indicated the total microbial biomass. The cumulative content of the following PLFAs was regarded as bacterial origin: i15:0, a15:0, 15:0, i16:0, 16:1ω7c, 17:0, i17:0, cy17:0, 18:1ω7c, and cy19:0. The PLFAs 16:1ω7c, cy17:0, 18:1ω7c, and cy19:0 represented gram-negative bacteria, whereas the PLFAs i15:0, a15:0, i16:0, and i17:0 represented gram-positive bacteria. The PLFAs 18:2ω6,9c, 16:1ω5c, and 10Me18:0 represented common fungi, vesicular arbuscular mycorrhizae, and *Actinomycetes*, respectively (Zogg et al., 1997; Zelles, 1999).

### DNA extraction, PCR amplification, and amplicon sequencing

Total genomic DNA was extracted from 0.25 g soil samples with the DNeasy PowerSoil Pro Kit (Qiagen, Hilden, Germany) based on the manufacturer’s instructions. Purified DNA was quantitated using the Qubit dsDNA HS assay (ThermoFisher Scientific, San Jose, CA, USA), and qualitated using the Agilent 4200 TapeStation system (Agilent Technologies, Palo Alto, CA, USA). The LoopSeq™ 16S Long Read Kit (Loop Genomics, San Hose, CA, USA) was used to generate the full-length 16S rDNA amplicon library. In particular, V1 to V9 regions were amplified by specific primers (27F: 5′-AGAGTTTGATCMTGGCTCAG-3′, 1492R: 5′-TACCTTGTTACGACTT-3′) and sequenced on an Illumina NovaSeq platform (Illumina, San Diego, CA, USA) using a paired-end 150 bp mode. The cloud-based platform maintained by Loop Genomics processed raw short-reads into assembled high quality consensus contigs. Following Callahan et al. (2021), those were further processed using DADA2 (Callahan et al., 2016), where amplicon error detection / correction, potential base error filtering, and chimeric sequence removal were conducted to obtain amplicon sequence variants (ASVs). Sample preparation, library construction sequencing, and data analyses were performed by Welgene Biotech Co., Ltd. (Taiwan, ROC). Shannon and Simpson index were calculated with vegan package in R v.4.3.0 program.

### Sequence data analyses

Taxonomy classification was performed using RDP Naive Bayesian Classifier algorithm (Wang et al., 2007), with minimum bootstrap confidence of 50 searching in the NCBI (National Center for Biotechnology Information) 16S ribosomal RNA sequences under RefSeq records of BioProject 33175 and 33317 (Haft et al., 2018) supplemented by RDP (Ribosomal Database Project) database (Cole et al., 2014), totaling to 987 taxa were classified.

### Statistical analysis

Data obtained from fresh soil samples were adjusted to an oven-dried basis by considering the soil moisture content. For between-practice comparisons, we performed a one-way analysis of variance and Duncan’s multiple range test. By using Canoco (version 5.0) for Windows, we performed redundancy analysis (RDA) to analyze whether microbial communities and soil enzymatic activity were associated with the following environmental factors assessed in our previous study (Lin et al., 2019): microbial biomass carbon, microbial biomass nitrogen, SOC, and TN. Statistical analyses, unless indicated otherwise, were performed using SPSS (version 18.0; SPSS Inc., Chicago, IL, USA). A *p-*value of < 0.05 was considered to be statistically significant. RDA is the multivariate (meaning multiresponse) technique analogue of regression.

Partial least squares path modeling (PLS-PM) was performed to identify the pathways though which the study variables including different management practices, soil erosion, bacterial abundance, phosphorus and nitrogen, regulate soil carbon stock contents under various agricultural management practices. The PLS-PM model was constructed using the “innerplot” function of the “plspm” package. The quality and performance of the model were evaluated through the goodness-of-fit test. The “ggplot2” package in R was used for RDA and plot generation (Villanueva and Chen, 2019).

## Results

### Soil properties and soil carbon stock

Soil samples collected from the orchards under OF and EFF had the lower pH than under CK (Table 1). SOC and the carbon-to-nitrogen ratio were significantly higher in OF soil than other soils indicate high input of organic C in OF soil. The TN content was lower in CF soil than in OF and EFF soils. However, no significant difference was observed between OF and EFF soils. The contents of total and available phosphorous were significantly higher in CF soil than in other soils. Unlike CF, OF and EFF resulted in low soil pH because of high organic matter content. Microbial biomass carbon reflects soil microbial activity.

**TABLE 1:** Chemical properties of soils and characteristics of microbial communities under various agricultural management practices.

Treatments	pH	Texture	OC (g kg^–1^)	TN (g kg^–1^)	NH_4_^+^-N (mg kg^–1^)	NO_3_^–^-N (mg kg^–1^)	TP (mg kg^–1^)	Av-P (mg kg^–1^)	MBC (g kg^–1^)
OF	6.67 a	SCL	46.8 a	1.40 ab	25.0 ab	24.0 a	610 b	21.0 b	1.52 a
EFF	6.03 b	SL	22.8 b	1.53 a	30.3 a	15.9 a	227 c	30.3 b	1.04 ab
CF	7.33 ab	SL	14.5 c	1.07 b	22.0 b	23.9 a	779 a	135 a	0.48 c
CF* (1975)	6.04	fSL	11.2	–	–	–	–	–	–

OF, organic framing; EFF, ecofriendly farming; CF, conventional farming; SCL, sandy clay loam; SiL, silty loam; SL, sandy loam; OC, organic carbon; TN, total nitrogen; NH_4_^+^-N, ammonia; NO_3_^–^N, nitrate; TP, total phosphorus; Av-P, available phosphorus; MBC, microbial biomass carbon. The soils were collected and analyzed in 2020. The letters after the values in each column indicate significant between-practice differences (*p* < 0.05).

*An earlier soil was surveyed and analyzed in the same studied area with the same fruit planting with CF practice based on Soil Survey Report published in 1975 by National Chung Hsing University.

**FIGURE 2:**
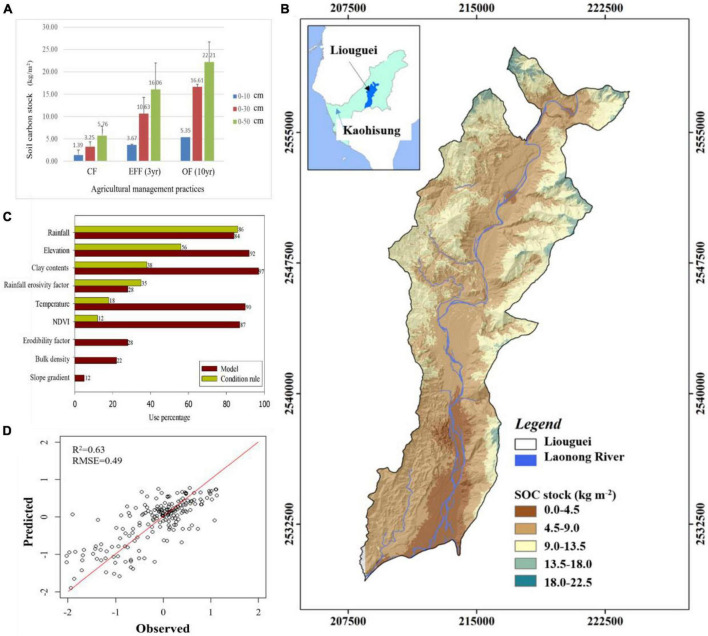
**(A)** Carbon stock (kg/m^2^) in soil samples collected from different depths in the orchards under various agricultural management practices; **(B)** relative importance of our selected environmental variables; **(C)** measured SOC stock (kg m^–2^) versus predicted SOC stock (kg m^–2^) via CUBIST model (black solid line represents the 1:1 line while the blue points are the model validation datasets); **(D)** prediction spatial distribution of SOC stock in Liouguei district in this study. CF, conventional farming; EFF, ecofriendly farming; OF, organic farming.

Our findings indicate that OF and EFF resulted in higher contents of microbial biomass carbon than did OF and CF. Regarding nutrient status, the content of TN and NH_4_^+^-N were higher in EFF soil than in other soils, suggesting a higher content of organic nitrogen in EFF soil. The contents of total phosphorus was higher in CF soil than in other soils, possibly because of farmers’ long-term use of chemical fertilizers. OF soil had the highest SOC stock. Our findings revealed that various agricultural management practices exert distinct effects on soil carbon sequestration and soil quality assessment in high-yield fruit-growing areas located in shallow mountainous regions within tropical climates. We compared soil carbon stock and organic matter quality between the agricultural management practices. The implementation of diversified farming practices (agroforestry) such as OF and EFF effectively increased the content of SOC stock. OF increased SOC stock contents by approximately 1.35 kg/m^2^/30 cm and 1.65 kg/m^2^/50 cm per year, whereas EFF increased it by approximately 2.45 kg/m^2^/30 cm and 3.40 kg/m^2^/50 cm per year (Figure 2A). The results indicated that EFF significantly promoted soil carbon sequestration, resulting in a net increase of soil carbon stock of 6.0 kg/m^2^ per year, which was 2.8 times higher than the value obtained for CF soil.

To further understand the impact of land use types and agricultural management practices on the distribution of soil organic carbon stocks, this study utilized a machine learning-based multiple linear regression approach to predict and simulate the spatial distribution of soil organic carbon stocks using environmental variables (Figures 2B–D). Our modeling displayed a well simulation results (r2 = 0.63, RMSE = 0.49) for SOC stock distribution and rainfall, elevation, clay contents, Rm, temperature and NDVI were best variables. The results could facilitate the development of optimal agricultural management strategies for regions with severe soil degradation based on the predicted results.

### PLFA biomarkers

Phospholipid-derived fatty acids serve as indicators of active living biomass and were reflected by management practice. The total PLFA content was the highest in microbes collected from OF soil (Table 2). The contents of PLFA biomarkers were significantly higher in OF soil than in other soils. Similar to patterns observed for the total PLFA content, the contents of both fungal and vesicular arbuscular mycorrhizal biomarkers were the highest in OF soil and lowest in EFF soil. The contents of PLFA biomarkers for gram-positive and gram-negative bacteria were the highest in OF soil. The ratio of gram-positive to gram-negative bacterial biomarkers significantly decreased from OF soil to CF soil. No significant between-practice difference was observed in the ratio of fungi to bacteria.

**TABLE 2:** Contents of phospholipid-derived fatty acid biomarkers in the total and individual microbial communities in soils under various agricultural management practices.

Treatment	Total PLFA (nmol g^–1^ soil)	Bacteria	Fungi	VAM fungi	Actinobacteria	G+ bacteria	G− bacteria	G+/G−	Fungi/bacteria
OF	17.51 a	7.66 a	0.38 a	0.73 a	0.66 a	4.76 a	2.64 a	1.80 a	0.05 a
EFF	11.93 b	5.32 b	0.15 b	0.50 b	0.46 b	3.01 b	2.12 b	1.14 b	0.03 a
CF	12.37 b	4.91 b	0.24 ab	0.59 b	0.39 c	2.75 b	1.96 b	1.43 b	0.05 a

PLFA, phospholipid-derived fatty acid; VAM, vesicular arbuscular mycorrhizae; OF, organic farming; EFF, eco-friendly farming; CF, conventional farming; G+, gram positive; G-, gram negative. Different letters after the values in each column indicate significant between-practice differences (Duncan’s multiple range test; *p* < 0.05).

### Soil microbial community structure

**FIGURE 3:**
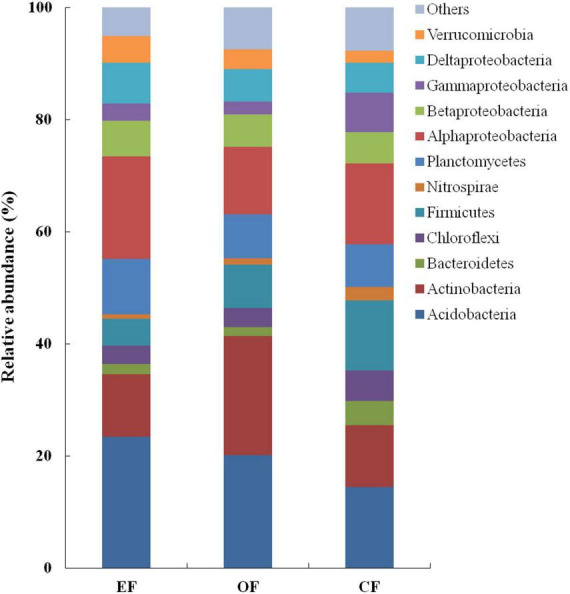
Relative abundances of bacterial communities under various agricultural management practices. CF, conventional farming; OF, organic farming; EF, ecofriendly farming.

In soils under the three agricultural management practices, the relative abundances of *Acidobacteria* (14.5–23.4%), *Actinobacteria* (11.0–21.2%), and *Proteobacteria* (25.9–35.2%) were the highest among bacterial communities (Figure 3). However, the relative abundances of other phyla, such as *Chloroflexi*, *Firmicutes*, and *Planctomycetes*, were low, constituting < 9.0% of all bacterial communities (Figure 3). Within the group of *Proteobacteria*, the relative abundances of *Alphaproteobacteria* (approximately 14.9%) and *Deltaproteobacteria* (approximately 6.2%) were the highest, whereas those of *Betaproteobacteria* and *Gammaproteobacteria* were the lowest (each constituting < 5.9% of all bacterial communities; Figure 3).

Despite observing similar abundances of bacterial phyla, we noted differences among the three agricultural management practices in the relative abundances of bacteria. In OF soil, *Actinobacteria* accounted for approximately 21.0% of all bacterial communities. However, in EFF and CF soils, this genus of bacteria accounted for only approximately 11.0% of all bacterial communities. *Proteobacteria* constituted 35.0% of all bacterial communities in EFF soil but only 26.0% of all bacterial communities in OF soil. Within the group of *Proteobacteria*, the relative abundances of *Alphaproteobacteria*, particularly those belonging to the order *Rhizobiales*, were higher in EFF soil than in OF and CF soils. *Firmicutes* accounted for 12.5% of all bacterial communities in CF soil but < 8% of all bacterial communities in EFF and OF soils.

### Association between microbial communities and soil properties

**FIGURE 4:**
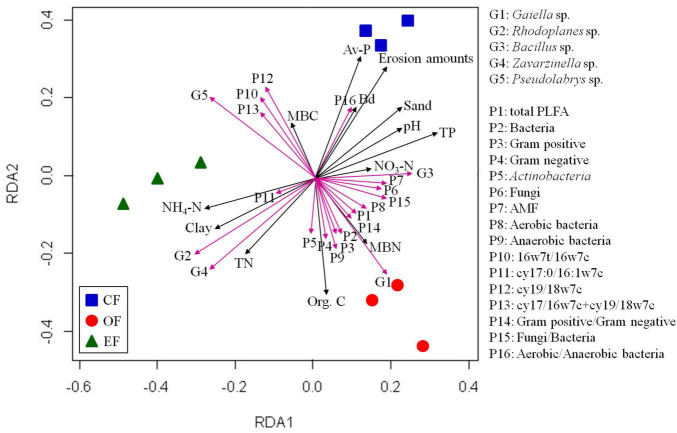
Results of redundancy analyses of soil properties and microbial communities under various agricultural management practices. CF, conventional farming; OF, organic farming; EF, ecofriendly farming.

We evaluated the association between soil microbial communities and environmental factors. The RDA of microbial communities and environmental factors revealed patterns (Figure 4). The contents of SOC, total phosphorous, and TN were positively correlated with the relative abundances of soil microbial communities, but that of available phosphorous was negatively correlated with such abundances. In summary, distinct physiochemical properties of soil and the contents of SOC, nitrogen, and available phosphorous influenced the development of bacterial and fungal communities in soils under various agricultural management practices.

RDA was performed to identify the associations between microbial communities, PLFA contents, and soil properties. On the basis of the results, three distinct bacterial clusters were identified in soils under different agricultural management practices (Figure 4). Positive correlations were observed between microbial communities and organic carbon and microbial biomass nitrogen contents in OF soil, between microbial communities and TN and NH_4_-N contents in EFF soil, and between microbial communities and pH and available phosphorus contents in CF soil (Figure 4). Furthermore, the relative abundance of *Gaiella* and the contents of PLFAs were positively correlated with the contents of SOC and microbial biomass nitrogen, as were the cumulative content of PLFAs and the abundances of gram-positive and gram-negative bacteria. The relative abundances of the genera *Rhodoplanes* and *Bacillus* and the content of the PLFA cy17:0/16:1w7c were positively correlated with the contents of TN and NH_4_-N. PLFA contents in aerobic and anaerobic bacteria were positively correlated with pH and available phosphorus contents (Figure 4).

**FIGURE 5:**
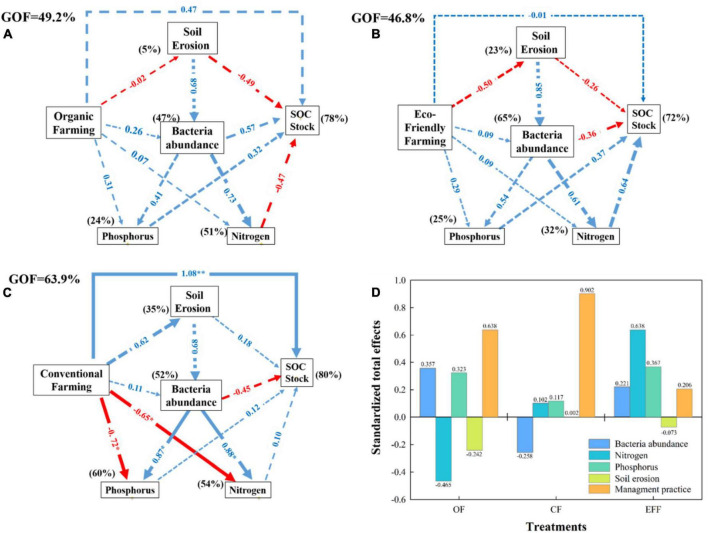
Partial least squares path modeling (PLS-PM) analysis of the relationships between management practices **(A)** organic farming; **(B)** eco-friendly farming; **(C)** conventional farming; **(D)** standardized total effects for all management practices, soil properties, bacterial abundance, and soil C stock. Standardized total effects of bacteria abundance, nitrogen, phosphorus, and soil erosion on soil C stock. Management practices include input amounts of C, N, and P. Soil erosion: Bd, clay, erosion amounts. Bacterial abundance: Shannon index, total PLFA, fungi, aerobic/anaerobic. Phosphorus: total P and available P. Nitrogen: total N, NH_4_^+^-N, NO_3_^–^-N. SOC stock: NDVI, SOC contents. A total of 1,000 bootstraps were conducted to estimate the path coefficients. Positive and negative effects are represented by blue and red arrows, respectively. Path coefficients that were not significantly different from zero are shown as gray dashed lines; *p < 0.05, and **p < 0.01. Percentages above the boxes represent the explanatory power of the variables. The goodness-of-fit was used to assess the model.

**FIGURE 6:**
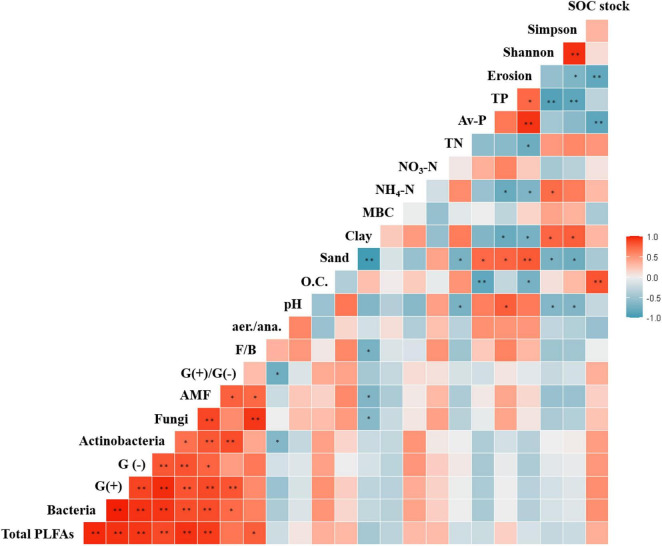
Pearson correlations between soil properties and agricultural management practices. *p < 0.05 and **p < 0.01.

The associations between management practices, soil properties, microbial diversity, and soil carbon stock contents were investigated through PLS-PM analysis (Figure 5). The period after transition from CF to OF or EFF was not sufficiently long to significantly (*p* < 0.05) affect all variables (Figures 5A, B). The long-term implementation of CF in the study plots might have significantly affected certain variables (Figure 5C). The three management practices improved soil carbon stock. However, a direct effect (*p* < 0.05) was exerted only by CF. OF and EFF non-significantly (*p* > 0.05) reduced soil erosion, which in turn increased soil carbon stock content. A significant negative correlation was observed between SOC stock and soil erosion (Figure 6). The three management practices positively affected bacterial abundance; however, the effects were non-significant (*p* > 0.05; Figure 5). The management practices affected nitrogen and phosphorous contents either directly or through their effect on bacterial abundance. As shown in Figure 5, OF and EFF non-significantly increased (*p* > 0.05) nitrogen and phosphorous contents, whereas CF significantly (*p* > 0.05) reduced these contents. Regarding standardized total effects (Figure 5D), the contents of carbon stock in CF, OF, and EFF soils were primarily driven by management practices; management practices, microbial diversity, and nitrogen cycle; and nitrogen and phosphorous cycles, respectively. The PLS-PM analysis explained 78, 72, and 80% of the total variances in the contents of carbon stock in OF, EFF, and CF soils, respectively (Figure 5).

## Discussion

In the present study, bacterial communities in soils under the three agricultural management practices were dominated by *Acidobacteria*, *Actinobacteria*, and *Proteobacteria*. These phyla have been demonstrated to be widely distributed across soil ecosystems under different management practices (Lupatini et al., 2017; Durrer et al., 2021). The relative abundance of *Acidobacteria* was lower in CF soil than in EFF and OF soil. This might be because of the negative correlation between this phylum and soil pH (Xiong et al., 2012), which was also corresponded with the results in this study (Figure 6). *Rhizobiales* was abundant in EFF soil. This order of the class *Alphaproteobacteria* includes members that can fix nitrogen, decompose organic compounds, and promote plant growth (Yarwood et al., 2009). Thus, these bacteria may help maintain soil function and indicate the effects of EFF on soil bacterial communities. In addition to the relative abundances of *Acidobacteria* and *Alphaproteobacteria*, that of *Actinobacteria* varied across the three management practices. Bacteria belonging to the phylum *Alphaproteobacteria* can degrade recalcitrant organic matter (Zhou et al., 2016) and are abundantly present in OF soil (Hartman et al., 2018).

### Changes in soil physicochemical properties in response to various agricultural management practices

Unlike CF, OF, and EFF significantly increased the contents of SOC and TN, indicating that the application of organic and ecofriendly fertilizers can increase the contents of SOC and TN. Higher application contents of organic fertilizers and herb biomass under the OF system might have resulted in the higher contents of organic carbon and nitrogen in OF soil. Similarly, the application of higher contents of herb biomass under the EFF system might have increased the contents of litter and humus layer in surface EFF soil, although the orchard farmers adopted the EFF system since 2018. This might have been because of the application of organic fertilizers under the OF system or the provision of additional nutrients under the EFF system, which might have increased the content of litter, thereby improving soil fertility.

Unlike OF and EFF, CF involved the use of herbicides, which altered the community structure of soil microbes. *Gammaproteobacteria*, *Planctomycetes*, *Firmicutes*, *and Nitrospirae* were abundant in CF soil; this might have been because of the regular use of phosphorous-containing herbicides (Giambò et al., 2021) and chemical fertilizers. Some members of the class *Gammaproteobacteria* are plant and animal pathogens; long-term application of herbicides in soil can promote the growth of plant pathogens (Pileggi et al., 2020). Long-term deficiency of SOC may lead to increased growth of *Planctomycetes* and *Firmicutes*, which can decompose fresh organic matter and thus alleviate nutrient deficiency stress (Liu et al., 2023). The effects of sublethal doses of a phosphorous-containing herbicide on honeybee microbiota have been reported by Giambò et al. (2021); herbicide exposure reduced the abundances of *Planctomycetes* and *Firmicutes*.

### Changes in the community structure of soil microbes in response to various agricultural management practices

Plants may regulate the contents of microbial PLFAs in soil (Priha et al., 1999). Changes in vegetation, rather than direct changes in soil properties, have been demonstrated to alter the community structure of soil microbes (Zornoza et al., 2009; Wang et al., 2022). The application of guano in soils under the OF system, which results in increased contents of nitrogen, may increase the productivity of herbs and thus facilitate the growth of soil microbes (Wang et al., 2022).

The cumulative content of PLFAs and the relative abundances of bacterial communities have been reported to be positively correlated with the contents of SOC and TN (Shahbaz et al., 2020). We observed a high cumulative content of PLFAs and high relative abundances of gram-positive and gram-negative bacteria in OF soil; this might have been because of the high contents of SOC and TN in OF soil. Although EFF increased SOC and TN contents, no significant difference was observed in the abundances of soil microbial communities between EFF and CF; this might be because EFF did not involve the provision of any additional substrate, which resulted in lower microbial biomass in EFF soil than in OF soil. Some studies have indicated that gram-negative bacteria flourish under substrate-rich conditions; however, under resource-limited conditions, slow-growing gram-positive bacteria were more abundant than gram-negative bacteria (Djukic et al., 2010; Fanin et al., 2019). Although no additional SOC was supplied under the EFF system, the ratio of gram-positive to gram-negative bacteria was the lowest in EFF soil, suggesting that this soil facilitated the growth of gram-negative bacteria.

The abundances of fungi and vesicular arbuscular mycorrhizae were the highest in OF soil. There were no significant differences in the ratio of fungi to bacteria among three practices (Table 2). The slightly low ratio of fungi to bacteria in EFF might indicate the content and composition of litter entering the soil because fungi were the predominant decomposers of recalcitrant present in litter (Allison et al., 2005) and bacteria are efficient competitors for the availability of labile substrates (Djukic et al., 2010). The low ratio of fungi to bacteria in EFF soil also indicated that ecofriendly agricultural management practices can facilitate the rapid cycling of carbon in soil. Fast-growing plant species, particularly those with highly branched fine root systems, release large quantities of exudates into EFF soil (Personeni and Loiseau, 2004), which are favorable for bacterial growth.

In this study, the RDA indicated that the improvement in soil quality through framing practices was directly associated with the changes in the community structure of soil microbes. Furthermore, OF positively affected the contents of both soil microbial biomass and SOC, suggesting that the carbon and nitrogen sources present in OF soil significantly increased the content of microbial biomass. The PLS-PM analysis revealed that the type of nutrients varied between CF and the other two agricultural management practices, which may explain the significant differences in the relative abundances of bacteria and the contents of nitrogen and phosphorous between CF and OF or EFF. Long-term application of chemical fertilizers under the CF system positively affected nitrogen and phosphorous cycles but negatively affected microbial characteristics (Figure 5). Tan et al. (2023) indicated that manure application leads to the formation a more complex co-occurrence network than does mineral fertilizer application (Ling et al., 2016); these findings are consistent with our findings (Figure 5). The aforementioned phenomenon may be attributed to the different nutrient characteristics of manure and mineral fertilizers. Mineral fertilizers release fast-acting, broad-spectrum nutrients, which help alleviate nutrient limitation stress and interspecies competition, thereby favoring many fungal taxa (Sun et al., 2020). By contrast, manures release narrow-spectrum nutrients, favoring the growth of only specific fungal species because of their differential utilization of carbon substrates (Hanson et al., 2000; Rinnan and Bååth, 2009). Thus, unlike mineral fertilizers, manures exert substantial selection pressure on soil fungi.

## Conclusion

In our conclusion, the bacterial communities of tropical slope lands depends on the management practices. The management practices of OF or EFF could not only effectively increase microbial communities but also promote enzyme activities. Furthermore, soil organic carbon stock has obviously increased at least 3–5 times by converting CF into EFF or OF. Nutrient cycling is also affected by SOC increasing in OF or EFF, which abundance of C also means C limitation alleviation and further resulted in N and P deficient in those soils. However, Facing climate change scenarios, including soil degradation and food security, our results provide valuable information for demonstration and comparing changes in soil bacterial communities and soil quality in cultivated slope lands under tropical climate.

## Data availability statement

The raw data supporting the conclusions of this article will be made available by the authors, without undue reservation.

## Author contributions

Y-TL and S-HJ conceived and finished the study, performed the analyses, drew all pictures, and drafted the manuscript. Y-MT contributed to methodology and supervision and wrote, review, edited the manuscript and provided a great contribution in the revision version. C-NC and S-HJ performed the research. E-HC contributed to sampling and water quality analysis. C-NC and S-HJ framed the manuscript and contributed to revisions. All authors contributed to the article and approved the submitted version.

## Footnotes

1
https://data.gov.tw/

2
https://cran.r-project.org/src/base/R-4/R-4.3.1.tar.gz
